# Is Radio-Therapy a Fad?

**Published:** 1905-02

**Authors:** 


### "IS RADIO-THERAPY A FAD?"

ATLANTA, GA., January 23, 1905.

DEAR DOCTOR: Referring to your editorial with above title, in January issue, radio-therapy is a fad in so far as it has become a spectacular source of revenue to so many men who have no further competence than the pocket-book to buy the apparatus. If men without knowledge of skin diseases and cancer; even, as we have heard, dentists are practicing X-ray therapy, it is a fad and a very dangerous one. In the hands of men competent to diagnose and treat these conditions in any indicated way, radio-therapy as you well know is a firmly established measure of the greatest utility. In the hands of the electrician or untrained practitioner it is capable of much injury both to the patient and, reactive, to its own value.

The object of this note is simply to emphasize the last part of your editorial and to give a warning as to the sources of discredit to radio-therapy. It is a treatment of too much value to be allowed to fall into disrepute through ignorant use.

Yours truly, —— ———.

[It was not the editor's purpose to throw discredit upon radio-therapy in its true sense. He is familiar from personal experience with its great value. But he desired that it be spared the opprobrium that would inevitably be attached to it if it were overstrained or claims made for it too extravagant and sweeping ever to be materialized.]

---

The Committee on Tuberculosis from the State Medical Society desires to collect all statistics possible on Tuberculosis from the various cities, towns and counties throughout the State. The Secretaries of the various Boards of Health are requested to fill out this blank and mail same to Dr. R. R. Kime, 509-11 Eng. Amer. Bldg., Atlanta, Ga.

Population........ ——— White ....... ——— Colored ....... ———

| | 1900 | 1901 | 1902 | 1903 | 1904 |
|---|---|---|---|---|---|
| Total deaths all causes.......................... | .. | .... | .... | .... | ... |
| Total deaths from Tuberculosis.................... | ... | ... | .... | .... | ... |
| White deaths " " .................. | ... | .... | .... | .... | .... |
| Colored " " " .................. | ... | .... | .... | .... | .... |

Name of Town.................................

.................................Sec. Board of Health.

---

Mr. Flanagan: "The top of the mornin' to yees! And how do the family be, Mr. Hooligan?"

Mr. Hooligan: "They be doing well, thanks to ye. Tim comes home from college with gr-reat knowledge—ye niver heard the likes of it! He do say that there be boogs iverywhere—in the wather we dhrink, in the air we breathe, in our ohn bodies, even. And they calls thim different names in different places. In Germany they be germicides, in Paris they be parricides, and in ould Ireland they be Mike-robes. Moind that, now!"

---

**This Journal and Atlanta Semi-weekly Journal one year for $1.25 cash in advance. No checks. Strictly to new subscribers.**

